# “It makes me feel so much safer”: Sexual and gender minority community perspectives on telehealth use and implications for future practice

**DOI:** 10.1371/journal.pone.0345296

**Published:** 2026-03-19

**Authors:** Jeffrey A. Wilhite, Lisa Altshuler, Alexa B. D’Angelo, Anna Raykov, Annabelle Abbadessa, Maryam Roosta, Karmen S. Williams, Christian Grov

**Affiliations:** 1 Division of General Internal Medicine and Clinical Innovation, Department of Medicine, NYU Grossman School of Medicine, New York, New York, United States of America; 2 City University of New York Graduate School of Public Health and Health Policy, New York, New York, United States of America; Meharry Medical College, UNITED STATES OF AMERICA

## Abstract

**Introduction:**

Telehealth has the potential to expand access to care, though barriers including insurance coverage, technology literacy, and personal preferences have been described since the pandemic induced uptick. Sexual and gender minoritized individuals (SGMs) face unique challenges which make telehealth a particularly promising care option. Regardless of population needs, research efforts looking at experiences and opportunities following multiple years of use are missing from the literature. Given that telehealth is here to stay, this study explores telehealth experiences, barriers, and preferences of SGM individuals 4 + years after rapid uptick to inform future efforts for the community.

**Methods:**

Participants were recruited from an observational cohort study of SGM individuals. Eligible participants consented to follow-up contact and had previously used telehealth. Semi-structured interviews (N = 21) were conducted between March and April of 2024, covering telehealth use, barriers, and future directions. Interviews were recorded, transcribed, and thematically analyzed using a directed content analysis approach informed by prior literature. Coding was completed iteratively by four authors, with themes refined through group consensus, resulting in final recommendations for improving telehealth practice and engagement among SGM populations.

**Results:**

The majority of participants were White (52%), cisgender men (90%) with at least some college education (90%), employed full-time (73%), and residing in the southern U.S (57%). Participants highlighted telehealth’s benefits, including provider accessibility, convenience, and comfort discussing sexual health needs with affirming clinicians. They found telehealth particularly useful for medication management, mental health, and sexual health services. They also emphasized the importance of promoting telehealth as providing privacy, security, and culturally competent care, findings which underscore their utilization motivators and shed light on unique needs of the population. Participants recommended improvement opportunities, such as creating/expanding directories of LGBTQ + -friendly providers and increasing/tailoring community-targeted marketing through social media and physical spaces.

**Discussion and conclusion:**

We interviewed a diverse group of SGM-identifying telehealth users to explore their experiences with telehealth-based care. Participants expressed ongoing interest in telehealth overall, but conversations highlighted future opportunities and provided clear next steps for research, practice, and marketing. Our findings provide valuable insight for the next era of telehealth use.

## Introduction

Telehealth, or synchronous remote-based clinical care (e.g., by telephone, video), has the potential to remove barriers associated with access, as decades of research suggest that remote-based clinical care can increase accessibility of services applicable to a patient’s health needs. In addition, evidence supportive of the effectiveness of telehealth to support a range of clinical conditions is strong [1–3]. Telehealth is not new to the field of clinical care, but rather an option which expanded exponentially during the early months of the pandemic era and has since gained a foothold as a common method of care [4]. Since initial expansion, utilization has varied by patient characteristics, including demographics like insurance status, race and ethnicity, and income level, and by reason for utilization. Recent analyses of 2022 data suggests that sexual minority individuals were more likely to use telehealth than their heterosexual counterparts during the early years of the pandemic era, and noted its convenience and ease of social distancing among their reasoning [5]. Supported by claims data in recent years, telehealth use has shifted primarily to primary care, urgent care, and mental health services, [6] each of which allow patients to access essential clinical care when necessary. In addition, coverage of telehealth services by insurers had largely been based on policies put in place prior to the pandemic era’s expansion of coverage, [7,8] with more affluent and medically necessary patients being approved for use [4].

Pandemic era telehealth research suggests that other barriers to uptake include insurance coverage, technology literacy of both patients and clinicians, and even preferences for care (e.g., preference for in person or telehealth) [9–11]. Though research has explored characteristics of users and their barriers in the broad, general population, less has been done to explore experiences of traditionally marginalized and underrepresented populations. Sexual and gender minority (SGM) individuals, for example, are a neglected, diverse community facing a range of health challenges falling outside of scope of their heteronormative counterparts’ needs. Among these are ongoing incidence of HIV/AIDS and disease prevention by way of pre and post-exposure prophylaxis (PrEP and PEP), higher suicide, alcoholism and drug abuse rates, and even challenges with gender affirming care access [12,13]. In addition to these challenges, this same patient population faces personal, interpersonal, and societal level stigmatization for their sexual practices, each of which impact comfort with and willingness to engage with clinicians [14]. Though telehealth uses and experiences may be similar across SGM and non-SGM person populations, research efforts are needed to document their similarities and dissimilarities. These efforts are more relevant than ever, as society has moved multiple years into the pandemic era and as perspectives on telehealth’s role evolves.

If we posit the notion that telehealth is a potentially transformative tool, as evidenced by the literature, then understanding opportunities for SGM communities may be critically important in ensuring healthy lives for all [1–3]. While the SGM community has been studied in various ways for many decades, research on their telehealth utilization and preferences for care is an emerging topic area. A majority of SGM population telehealth studies have focused on single issues like access to gender affirming care over telehealth, or deployment of remote-based sexually transmitted infection (STI) screening [15–18]. Given the relative lack of research on the intersection of telehealth use among diverse profiles with varying sexual preferences and gender representation, explorations within the SGM community can shed light on preferences for care and opportunities for improvement and outreach. With these details in mind, we sought to explore experiences with telehealth since the early pandemic era in a pool of SGM individuals, with the goal of (1) identifying which barriers and challenges exist following multiple years of use and (2) describing their current utilization preferences. This was done in order to develop a series of recommendations for opportunities for improvement across clinical and outreach efforts.

## Methods

### Participant recruitment and data collection

Participants were recruited from a U.S. national observational cohort study of SGM persons who have sex with men [19,20]. Participants in the cohort were contacted via email (from March-April 2024) to take part in the current study on a first-come first-served basis. During outreach, participants of the former observational cohort study were offered an opportunity to join the study if they had consented to follow up recruitment opportunities and if they had participated in a telehealth visit at any point prior to recruitment. Three batches of 15–20 potential participants were contacted prior to recruiting the total number of participants in this study. Participants were provided a consent form over email and were asked to review the form and signify their willingness to participate prior to scheduling an interview with study personnel. At the start of the interview, participants reaffirmed their interest in participating, during which the consent guide was reviewed verbally and participants repeated IRB-approved consent statements (e.g., willingness to participate and to be recorded). An interview guide, consisting of 16 prompts (Appendix 1 in S1 File), was used to guide discussion on a series of topics, including uses and experiences, ongoing barriers, and future directions for telehealth. The first section, on uses and experiences, focused on discussions of participants perceptions and describing recent use cases (and frequency), and sharing perspectives on utilization. The middle portion of the interview consisted of questions surrounding barriers and overcoming challenges. The third and final portion focused on perceptions of the impact of telehealth on health outcomes, on conditions where telehealth is most and least useful, and on opportunities for increasing engagement and attractiveness to the SGM community.

Interviews were conducted on Zoom by lead author (JW). They were recorded and transcribed verbatim. Transcripts were then verified by comparing the original audio file to the transcript before being organized and analyzed for themes using Excel. Participants received a $40 gift card for participation. Interview length varied between 20 and 40 minutes, with 26 minutes being the median interview length. A total of 21 interviews were conducted from March-April of 2024 in order to capture the full breadth of telehealth user perspective and reach saturation (e.g., repeat themes and commentary) during conversations. This study was approved by City University of New York’s Institutional Review Board.

### Data analysis

During analysis, a directed content analysis approach was used to explore data captured in the interviews with participants [21]. Though presented as flexible in nature, a directed approach to content analysis takes into consideration the a priori assumptions of data as informed by prior literature review, analysis, or publication review. A directed approach was chosen given the objectives of the current study, which include the goal of describing user experiences across core areas noted in the guide, and necessary transparency surrounding author overlay of experiences and perceptions onto data during analysis phases.

The analysis phase took place iteratively, over a series of several weeks. During the first phase, four authors (JW, AR, BA, MR) coded data using an a priori codebook developed by lead author (JW). This codebook was directly informed by a literature review of former reports of telehealth barriers and experiences, aligning with question prompts from the interview guide. An additional open-ended section was added to the Excel codebook to capture any codes and themes falling outside of the scope of the items in the interview guide. During analyses, coders met weekly to review and discuss challenges with analyses, review specific quotations and coding, and to resolve issues through consensus. Following the first round of coding, the authors convened to review, edit, and merge codes. A final series of codes with representative, emergent themes were then developed. Themes were then reviewed and refined (both broadly and by demographic grouping), and a series of recommendations were generated.

## Results

A total of 21 participants were interviewed. Participants were primarily White (52%, N = 11), had some college level education or above (90%, N = 19), were employed full time (72%, N = 15), and were located in the southern United States (57%, N = 12) (Table 1). Nearly all, 90% (N = 19) identified as cis male while the remainder identified as trans female or “other”. Fifty-seven percent (N = 12) had an income level under $50,000 annually. With regard to insurance status, 72% (N = 15) held private insurance, 14% (N = 3) held government/public insurance, and 14% (N = 3) were uninsured. At the time of the interview, the age of participants ranged from 25–53 (median age = 38). There were no significant differences in terms of demographic information between interview participants and the larger survey sample from which they were recruited.

A series of themes, each with underlying sub-themes, emerged during the analysis phase, including views of telehealth, ongoing barriers, and opportunities (Appendix 2 in S2 File). These themes contributed to development of a series of recommendations.

**Table 1:** Demographics of participants.

Item		N (%)
Age Range	25-53	21 (100%)
Race	Asian	1 (5%)
	Black	6 (29%)
	White	11 (52%)
	More than one race	2 (9%)
	Unknown/Not reported	1 (5%)
Ethnicity	Latino	7 (33%)
Gender	Male	19 (90%)
	Transgender female or Other	2 (10%)
Education Level	HS or less	2 (10%)
	Some college or associates	9 (43%)
	4-year degree and above	7 (33%)
	Grad school and above	3 (14%)
Income level	$49,999 or Less	12 (57%)
	$50,000 +	9 (43%)
Insurance status	Private	15 (72%)
	Government/Public	3 (14%)
	Uninsured	3 (14%)
Employment status	Full time	15 (72%)
	Part-time	2 (9%)
	Unemployed	4 (19%)
Region	West	3 (14%)
	South	12 (57%)
	Northeast	3 (14%)
	Mid-West	3 (14%)

### Views of telehealth

A few notable constructs emerged under the view of telehealth theme, including benefits of use and preferences for care. Participants noted that accessibility of providers, comfort and convenience were benefits. Telehealth was described as removing barriers of traditional care methods, including selection of specific doctors based on their characteristics (i.e., openly gay clinicians), and allowing users to feel safe when sharing information about their sexual health needs as SGM persons. For example, one participant said “*I’m much more willing to reach out to people and ask questions over the phone or on video. It’s just a lot easier to access.” –* Participant 17 (40-year-old, White). Participants also noted a preference for medication management and non-emergency services over telehealth. Specifically, participants described medication refills, mental health, and sexual health needs as three promising areas for telehealth use as part of their care.

Complementary to their practical descriptions of experiences and views, details given by participants described latent themes surrounding psychological safety/trust in health care and relationship building with clinicians (Table 2), both of which were described as being facilitated through telehealth and of great importance and relevance to the SGM community. Though commentary reflective of the role of psychological safety was most commonly discussed by participants residing in southern states, quotations reflective of its importance were shared by participants from each demographic subgroup.

**Table 2:** Motivators within the SGM population.

Underlying constructs	Sampling of representative quotes	Implications
Psychological safety, privacy, security (potential to create a safe space via telehealth) Reduces judgement and improves communication Potential motivators of use (preference for telehealth)	*I have a queer, non-binary mental health therapist, which I’ve never had the opportunity to have in my life, and it’s made it so effortless to have a conversation…and it makes me feel so much more validated…It makes me feel so much safer.* - Participant 3 (32-year-old, White) *They wouldn’t have to worry about any private information coming out or getting out, or anybody else overhearing. So yes, time saving, privacy and discretion would definitely be the top selling points.* - Participant 16 (39-year-old, Multiracial) *If there were organizations [advertised] that are specifically dedicated to LGBT health care, where you can go and schedule some tests or talk [with] someone about PrEP, that would probably make [telehealth] more appealing.* - Participant 2 (32-year-old, Asian)	• SGM population is likely using telehealth for safety/privacy and they see that telehealth could be beneficial to other community members for the same reason. • Telehealth creates a space for the community to get their health needs met without fear.
	*If you don’t have any doctors that [are] necessarily specialized in LGBT health care, or maybe you’re in an area where there’s stigma around being LGBT, being able to find a healthcare provider that’s within the same state but is more comfortable with LGBT people. I think that would be helpful and making the participant a little bit more comfortable [with telehealth].* - Participant 3 (32-year-old, White) *A better directory of LGBT friendly doctors that I can connect to easily. Just having some kind of way of filtering that out…these are people who are comfortable talking about gay issues.* - Participant 17 (40-year-old, White) *….maybe skills of the therapist which is ensuring that your client feels heard or respected, and that the client feels trust in the provider, and that they have the skills appropriate to handle what you’re talking about.* - Participant 14 (28-year-old, Black)	• Potentially allows some SGM patients to feel more seen and heard. • Reinforces the notion that telehealth remains relevant for this community, and expansion/policy efforts would be beneficial for ensuring access.
	*It could allow somebody [to]….get access to care like PrEP or other things that they maybe are too embarrassed to go to their family doctor to see about getting, and so it could provide an a more accessibility. To…feel more confident, and [use] their visit to get the things that they need for their own health that they’re too, embarrassed to ask about.* - Participant 11 (44-year-old, White) *If you have STD symptoms you would like to [keep] private and you don’t want anybody [to] see you at the doctor’s office, [telehealth] is safe. So, [telehealth is the] way to be a little bit private about the treatment or the diagnoses.* - Participant 1 (40-year-old, Latino) *I would say personalization, because we’re people that have dynamic issues. And to that point, my mental health provider allowed me the ability to align with [a clinician] with my identity. And a lot of [other] providers can be doing that.* - Participant 3 (32-year-old, White) *It also provides opportunities for people who normally wouldn’t want to see a counselor or don’t have the ability to see a counselor or psychologist.* - Participant 2 (32-year-old, Asian)	Further research is needed, including gathering of additional perspectives of users *and* non-users in the community.

*I have a queer, non-binary mental health therapist, which I’ve never had the opportunity to have in my life, and it’s made it so effortless to have a conversation…and it makes me feel so much more validated…It makes me feel so much safer.*

- Participant 3 (32-year-old, White), describing accessibility, trust and safety, and relationships

*It [telehealth] could allow somebody who doesn’t have confidence in local doctors [to] get access to care or medicine like PrEP or other things that they are too embarrassed to go to their family doctor to see about getting…To remain not really anonymous, but feel more confident, and [use] their visit to get the things that they need for their own health that they’re too embarrassed to ask about.*

- Participant 11 (44-year-old, White), describing accessibility, trust and safety, and relationships

### Ongoing barriers

On the topic of challenges and barriers, participants commonly described challenges with timeliness of visits, with patient-physician interactions (underscoring the importance of relationships again), and ongoing issues with technology. Timeliness issues included overscheduling and the inability to schedule an appointment quickly, both of which led to delays with seeing a doctor. Overscheduling was cited as causing multiple-hour long delays, during which the participant was stuck in a virtual waiting room whereas scheduling challenges were described as being linked to a lack of continuity in systems where clinicians were assigned at random to provide telehealth care.

*…a lot of the times my provider, unfortunately, is back-to-back in meeting[s]. So if his previous meeting goes over, I’m sitting there on camera while I’m on break for work just waiting. And it’s eating up into my time.*

- Participant 3 (32-year-old, White), describing issues with timeliness of an encounter

*…it takes 3 to 4 months to get an appointment. I found myself reaching out to the telehealth medicine practices attached to different insurance companies. The problem with that is there’s no continuity of care, because they’ll just assign you whatever physician is available. I mean, they’ll see their notes…it’s kind of repetitive because you’re really not building the patient doctor relationship.*

- Participant 6 (53-year-old, Latino), describing issues with patient-physician interaction/communication as a result of less continuity

A smaller portion of participants described challenges with access. Among these were geographical barriers like only being able to access services in the state of employment or residence, clinicians offering telehealth services, and challenges with coverage of telehealth services under their insurance. One transgender participant, for example, noted they had challenges with consistently accessing their hormone treatments due to physician requirements (e.g., specific physicians not offering those services or requiring in-person visits for their transgender patients) and transportation issues, areas they eventually addressed through telehealth. They noted that telehealth can fill this same gap for others.

*...telehealth is for anything that’s routine. If you’re going in for hormones there’s blood work that’s sometimes done but the rest should be a little bit more of a routine process during quick check ups...its not really something that you need to have too much of a visual representation. You can update them on how you’re feeling and stuff, so that [has] worked out perfectly over telehealth for me.*

- Participant 20 (25-year-old, Latine), describing challenges and alleviation through telehealth use

### Opportunities

Under the opportunities domain, participants provided insight into systems level issues that require intervention. They also recommended expansion of offerings to the community, and provided insight into engagement and marketing opportunities to expand telehealth use.

### Systems issues

Among systems issues, participants noted perceived workflow issues and the need for additional resource allocation prior to visits. They described issues with scheduling that should be addressed so users aren’t waiting too long, along with an interest in having access to a list of clinicians that are similar to them and recommended offering technology to users if helpful.

*Not everyone has access to a laptop or a cell phone so possibly places where someone could go to have a telehealth appointment that’s still private, but is available to everyone.*

- Participant 11 (44-year-old, White), describing access assistance for some patients

### Service, marketing and advertising

Under the same domain of recommendations, interviewees noted the importance of targeting users through marketing and outreach efforts that meet the community where they are, whether that be in a club, on transit, or on their phone applications. They described using social media marketing and distributing/posting materials in community spaces where SGM individuals congregate. Though these recommendations were consistent across demographic subgroups, one participant outlier who was older in age (aged 53) recommended using radio for outreach. This same participants noted the importance of teaching digital literacy to elder SGM individuals.

*…apps…bars and clubs. Spaces like that, where people are going to actually see it and engage with it. I’ve been standing at urinals at bars, and I see things and I’m like, Hmm! I’m gonna check that out.*

- Participant 5 (28-year-old, Black), recommending locations for marketing

*Almost everyone is on some sort of app, whether it’s Facebook, Instagram, Scruff, Grindr…having the advertisements there gets my attention.*

- Participant 11 (44-year-old, White), describing use of apps for advertising

In terms of specific content included in marketing, participants expressed less desire for influencers to appear in advertisements and more interest in seeing diversity in marketing of telehealth to the community. In line with commentary on their views for telehealth, participants described the privacy, security, and non-judgmental clinicians in advertising of telehealth to the community; these were seen as important elements of both marketing and clinical care, reinforcing underlying notions of safety through telehealth use.

*I’ve seen a couple of ads recently. Better health had done a celebrity ad on Facebook. I don’t exactly know who it was, but it was a personal story, a paragraph of text about them.*

*-* Participant 3 (32-year-old, White), sharing details of memorable advertisements

*Showing diversity of our communities. Because it’s not all 25-year-old white boys. And something that shows the diversity of what’s offered by telehealth. Letting people know that it’s not just for your primary care or for PrEP.*

- Participant 11 (44-year-old, White), detailing selling points for engagement

*They wouldn’t have to worry about any private information coming out or getting out, or anybody else overhearing. So yes, time saving, and privacy and discretion would definitely be the top selling points.*

- Participant 16 (39-year-old, Multiracial), discussing privacy/discretion, underscoring underlying themes of safety through telehealth

Participants also described a general desire to see more active clinicians who either identify as and SGM persons or who specialize in SGM population care, along with more mental health offerings for the community.

*…if you’re a part of the LGBT plus community, and you don’t have any doctors that necessarily specialized in LGBT health care, or maybe you’re in an area where there’s a certain stigma around being LGBT, being able to find a healthcare provider that’s within the same state, but is more comfortable with LGBT people, I think that would be helpful.*

- Participant 2 (32-year-old, Asian), detailing the need for relatable providers, underscoring safety via telehealth

*Having more providers that identify as like LGBT + , I think that would help.*

- Participant 21 (31-year-old, Black Latino), noting a need for more SGMclinicians

## Discussion

We interviewed a pool of SGM individuals who use telehealth to capture their telehealth experiences at present (four years since the onset of the pandemic). Findings provided insight into preferences and ultimately informed a series of recommendations for the future of telehealth-based clinical care provision for the community (Appendix 2 in S2 File). Themes derived during conversations included ongoing barriers with regard to access, insurance coverage (even following expansion) and ease of use, preferences for specific services over telehealth, and opportunities for improvement, a theme with a distinct series of subthemes related to advertisement strategy, and workforce enhancement opportunities. Though practical in nature on the surface, these findings were underscored by descriptions of experience highlighting user concerns with psychological safety and relationship development/communication for SGM individuals accessing healthcare. Beyond implications for clinical practice and marketing efforts, these findings illustrate potential motivators for telehealth utilization within the SGM population. Key recommendations identified include ensuring users (including patients, staff, and clinicians) are prepared for visits (e.g., through workflow adjustments or resource allocation), deploying a more diverse, SGM population-specialized clinician pool, meeting users where they are in the community in terms of marketing, and editing advertisements to more effectively target SGM communities.

On aggregate, participants in our study expressed an ongoing interest in telehealth, and even noted a desire for expanded offerings. They described the ease with which telehealth can provide access to care, particularly for medication management (for trans- and non-transgender individuals alike), mental health, and non-emergency situations (i.e., sexual health, primary care). These data mirror the broader literature, much of which describes facilitating health care access as a key benefit of telehealth [22–26]. Findings from the current study also reflect trends in use since the initial uptick in telehealth integration, as primary care and psychiatry have been, and remain, the most commonly accessed services, per claims data [6]. Though participants in our sample described their desire for future telehealth opportunities, insurance-related challenges were noted, including issues with geographic location (i.e., residence state vs. working state, lack of multistate licensure) and finding a provider who is willing to offer telehealth. Challenges like these have been identified in prior publications, [27,28] but may become more relevant in the coming years if regulations fail to extend access. While Medicaid and Medicare-based telehealth coverage expanded in 2020, protections are only guaranteed through the end of 2024, [7,8] making the future of telehealth coverage uncertain for many patients. This potential loss of coverage can create a new access barrier for vulnerable patients. Further, access could become an even more pressing topic for patients who are especially vulnerable, like those who are transgender-specific therapies, as government policy and social perspectives fluctuate. Working to remove these barriers through lasting policy change will positively impact access for both SGM communities and their heterosexual counterparts.

Given the interests expressed by our sample and the relevance of access to the population, a key selling point of telehealth may be its use for ongoing care for sexual health, including PrEP, screening for STIs, maintenance of hormone treatment, and for accessing counseling and psychiatry services. A few participants also noted its use for non-emergency issues, such as a cold or similar illness. Though particularly relevant to the SGM person community, these types of services are useful to broader populations and working with policymakers and community members to sell these services as appropriate for telehealth can influence utilization rates, coverage regulation and even individual health statuses. Overall, our sample shared a positive outlook on telehealth as a clinical tool that should remain available, and a number of participants shared interest in expanding both services offered and the size of the pool of SGM population-focused clinicians. Development of the clinical workforce, and creation of tools (e.g., applications) to help SGM (and even non-SGM persons) patients link with clinicians more clearly supportive of their individual lifestyles can enhance care and entice new users.

Interestingly, we captured some underlying constructs during interviews, each of which appear to reflect care preferences and motivation for telehealth use (Table 2). Multiple participants described telehealth as capable of bolstering feelings of safety or trust in the healthcare system and in their clinician. Though discussed by participants from each demographic subgroup, those based in southern regions shared quotations supportive of the theme at the highest rate. Participants across the sample described telehealth as allowing less confident SGM individuals to feel both protected and discreet while accessing care, a notion underscoring the importance of psychological safety in healthcare both broadly and for the SGM community. Psychological safety is a critical component of patient-clinician relationships and is believed to enhance patient engagement and shared decision-making, both of which influence health outcomes [29,30]. At its most basic level, psychological safety influences the degree to which a patient is willing to open up to their clinician [29,31]. Given the disproportionate impact of certain health conditions, stigmatization, and policy on the SGM population [12–14] (e.g., issues becoming even more prevalent in the current political climate), psychological safety appears especially important to these patients. Though the relevance and importance of psychological safety in healthcare was reinforced by the entire sample, the higher propensity of commentary on privacy and safety from southern-based participants could be reflective of challenged with social norms in the region. Refining our interview guide and engaging in follow-up conversations could provide additional insight into unique needs of SGM individuals in the south. Ultimately though, ensuring the entire community has access to clinicians who make them feel safe is of grave importance. This importance was further reflected in quotations on participant interest in expansion of SGM-based services and clinician pools over telehealth.

Working across the board to ensure that telehealth remains available for SGM individuals is a clear next step, as are deeper, qualitative explorations of psychological safety for SGM individuals (both in telehealth and in-person settings, and among those in the southern United States) and their motivations for using or not using telehealth. These efforts can be done through legislative processes, including scaling up resources or ensuring that SGM communities have access to telehealth regardless of their insurance coverage (an issue also noted by our participants). Additional research in this area with both users and non-users to corroborate and expand findings would provide further insight on some of the constructs highlighted by our findings, ultimately enhancing the quality of our recommendations. Though potentially unsurprising given societal and political pressures placed on the community, our findings provide initial insight into motivators in our population and, when taken in combination with engagement efforts described by participants, provide a clear call to action for clinicians and policy makers alike.

Participants provided insight into advertising strategies and preferences. Our sample discussed the importance of outreach through multiple channels, including smartphone applications, pamphlets, and even public transit advertisements. In addition to this, they recommended advertising in the places where the community most commonly congregates, whether that be in community centers, or clubs/bars. One older participant (aged 54) explicitly described increasing frequency of advertisements on the radio. Given there were no stark differences in terms of recommendations based on demographics otherwise, it is difficult to unpack this recommendation without further data collection (potentially with elder SGM individuals specifically). Until future efforts provide more insight, a broad approach to outreach using multiple channels of communication is worthwhile. In terms of the literature, these locations may indeed be best for presenting messaging and outreach. Former efforts suggest, for example that social media advertisement leads to a reduction in STI transmission rates [32]. In addition to this, quality advertising can impact engagement in healthy behaviors [33]. From a design perspective, content of health advertisements should be tailored to both engage and meet the needs the target community [34,35]. Our findings run parallel to this notion, as participants shared an interest in seeing advertisements showcasing “normal” people using telehealth, whether that be through diverse SGM influencer marketing or user testimonials. Given our population appreciates the perceived safety that comes with telehealth use (reflective of the underlying construct of psychological safety); privacy, discretion, and ease of access for things like mental health and primary care/sexual health are marketable selling points that should be taken into consideration in future engagement efforts (and were described as so by participants). Given the potential for stigmatization burden present for SGM individuals during clinical encounters, reinforcing the discretion that comes with telehealth can impact engagement, and potentially health outcomes.

Finally, though multiple years into the post-COVID pandemic era, participants described some challenges reflected in the broader literature on telehealth that remain present today. Among these were technological/connectivity issues, clinician patient rapport/communication challenges, and visit timeliness. Both technology and rapport barriers have been described as concerns of patients and physicians previously [9,27]. Given the unique needs (e.g., discretion, safety, ongoing care) and health concerns of SGM individuals, rapport building/communication over telehealth appear particularly important to SGM individuals and highly relevant to their care quality, engagement, and health outcomes. From a training perspective, rapport building over telehealth is a challenging area that several training programs have begun to address [36,37]. Exposing entire members of the care team to training on virtual communication skills and practice interacting with SGM individuals will be beneficial for patient experience and care quality, and for building a safe space. Study of care team experience could fully unpack the root causes of barriers and inform/refine training and practice opportunities. In the interim, front or back loading a clinician’s schedule with telehealth visits (rather than a mix of in-person and remote visits littered throughout the day) could alleviate the backup of patient encounters, as could establishing a clear and consistent protocol for virtual care can enhance patient experience when a long wait time is present.

The findings of this study should be understood in light of their limitations. Given the nature of qualitative research efforts, the propensity to overlay analyst ideals onto findings is common. The lead author, for example, is a male-identifying researcher with a PhD-level education with an interest in technology use in healthcare. We attempted to address potential biases through reflexivity, use of four coders from diverse backgrounds, and group consensus during analysis. In addition, sampling bias can impact the validity of our findings. Participants in our study were volunteers with prior telehealth experience who opted to join the study and share their experiences. Future efforts should compare those who have used telehealth with those who have not used telehealth. This would provide more comprehensive recommendations for outreach and provide new context on psychological safety for SGM individuals. We attempted to address this limitation through recruitment of a study population from diverse demographic and socioeconomic backgrounds, and by reaching saturation point before completing interviews. Our sample also featured a wide range of ages. Regardless, recruitment of a larger or more diverse-in-experience sample could be useful in future efforts. Recruiting a diverse sample of transgender individuals, for example, could provide new insights not captured in the experiences of our small subset of transgender participants. Though there were no differences in commentary based on race/ethnicity, recruitment of an even more diverse sample in future efforts could give additional insight. Finally, interviews were conducted solely by the lead author. Though beneficial for creating a safe space for participants to share experiences, use of one sole interviewer may create the risk of interviewer bias.

## Conclusion

Throughout these interviews, we identified consistent challenges and opportunities for SGM telehealth users, identified their preferences for care, underlying motivators for utilization, and explored opportunities in which telehealth outreach can be expanded. Each of these findings has the potential to contribute to the health and livelihood of our population. Ultimately, working to remove barriers to access that remain present even after multiple years of use may be the first logical step. We believe our findings to be relevant to the broader research community, as they highlight opportunities for policy, clinical, and population level efforts. These findings can provide insight into successful integration of telehealth into clinical care provision for both SGM and non-SGM communities alike.

## Supporting information

S1 File Appendix 1. (DOCX)

S2 File Appendix 2. (DOCX)
