# Supplementary material for: “It makes me feel so much safer”: Sexual and gender minority community perspectives on telehealth use and implications for future practice
Source: PLoS One. 2026 Mar 19;21(3):e0345296. doi: 10.1371/journal.pone.0345296 (PMC13001967; doi:10.1371/journal.pone.0345296)
Supplement: S1 File — (DOCX) [file pone.0345296.s001.docx]

# **Appendix 1. Interview Guide**

***Conceptualizing use and experience since COVID began***

- To start, how would you describe telehealth to someone who’s never used it before?
- Follow up: If they describe, affirm that their description paints a great picture. If not, provide a conceptual description of telehealth.
- How did you first hear about telehealth as a tool for healthcare?
- If describing external influence – probe for details on how they heard

***Describing most recent visit – “tell me a little about your most recent encounter”***

- I’m hoping to get some details on your use since COVID began, so how often have you used telehealth since then?
- What do you typically use it for?
- I’d like to ask some questions about your most recent experience. How recent was it?
- Who was the encounter with? What medical condition was the encounter for?
- How did your clinician address your health condition during the visit?
- Can you describe the outcome of the visit?
- Would you describe it as a positive or negative experience?
- Follow up: What made this a particularly positive or negative experience?
- How has your use of telehealth changed (if at all) since the COVID period began?
- Did you, or have you ever, interacted with someone other than your primary clinician during a telehealth visit?
- If ‘yes’ – who did you interact with? Describe the interaction
- If ‘no’ – confirm they’ve only interacted with their primary clinician and skip to next question

***Barriers to use – “I’d like to ask a few questions about barriers or challenges you’ve encountered when using telehealth”***

- What sort of barriers, if any, have you experienced with accessing telehealth?
- What was challenging about these situation?
- How, if at all, have you resolved issues?
- Do you feel your needs are being met when you use telehealth?
- Any challenges in terms of interactions with the care team?
- If so, describe
- Can you describe your level of comfort with technology?

***Future directions – “Where can telehealth go from here for the gay community?”***

- How, if at all, has your view of telehealth changed over time?
- What impact can telehealth have on health outcomes?
- If confused – provide description of “health outcomes”
- Based on your experiences, what types of health conditions or use cases would telehealth be most useful for?
- Follow up: How about lease useful for?
- How often will you use telehealth in the future?
- If limited or won’t use again: What would increase your willingness to continue using this type of care modality?
- If regular: Any recommendations for improvement?
- Based on your experience, what do you think would make the option more attractive to other people in the gay community?
