# Supplementary material for: “It makes me feel so much safer”: Sexual and gender minority community perspectives on telehealth use and implications for future practice
Source: PLoS One. 2026 Mar 19;21(3):e0345296. doi: 10.1371/journal.pone.0345296 (PMC13001967; doi:10.1371/journal.pone.0345296)
Supplement: S2 File — (DOCX) [file pone.0345296.s002.docx]

| **Appendix 2. Practical recommendations gathered through qualitative interviews** | | | |
| --- | --- | --- | --- |
| **Prompt** | **Exemplary Representative Quote** | **Theme** | **Recommendations (from themes)** |
| Barriers you've experienced since beginning use? | There have been a couple of times where the link they've sent me doesn't work or the link is broken, or it just wasn't scheduled properly for whatever reason…then I have to sit there and wait for them to resend me the link.   - Participant 2 (32-year-old, Asian) | **Barriers to use** | - **Ensure that clinicians and patients are prepared before visit, that communication channels are tested, and that backup plans are prepared.** - **Ensure that scheduling is efficient and integrated into clinical workflow.** - **Ensure that physicians/clinicians are prepared (trained to do comprehensive telehealth visit), and work to increase care continuity in a clinic.** - **Work to change legislation and remove barriers associated with geography or treatment.** |
|  | I've had an instance where the program itself I had trouble connecting, so I ended up having to reschedule the visit. But it was the computer system, I'm not sure what, no one explained what it was that was happening. And the reason why it didn't connect in the moment [is unclear].   - Participant 11 (44-year-old, White) |  |  |
|  | The older generation, I think some of them need someone to help them understand how to use telehealth and get them set up doing it, cause it's it can be confusing.   - Participant 19 (53-year-old, White) |  |  |
|  | …a lot of the times like my provider, unfortunately, is back to back in meeting[s]. So if his previous meeting goes over, I'm sitting there on camera while I'm on break for work just waiting. And it's eating up into my time. And then during the pandemic I had a lot of issues where the provider would call for Tele Doc, and it would be hours on end.   - Participant 3 (32-year-old, White) |  |  |
|  | One time I called about an ear infection…and he told me to basically wait it out. I ended up having to schedule another doc, another virtual health visit to speak to someone before I could actually get services. While I could have gotten the medication that I needed on that [first call] I ended up having to wait 4 days to speak to another doctor in order to actually get support.   - Participant 5 (28-year-old, Black) |  |  |
|  | My only barrier is figuring out ways that the doctor can more adequately assess you without being in front of you.   - Participant 5 (28-year-old, Black) |  |  |
|  | I've had a hard time finding a primary care doctor here that is not part of a large group…it takes 3 to 4 months to get an appointment. So I found myself reaching out to the telehealth medicine practices attached to different insurance companies. The problem is there's no continuity of care, because they'll just assign you whatever physician is available plus you end up repeating [information]. I mean, they'll see their notes, but you know…it's repetitive because you're really not building the patient doctor relationship.   - Participant 6 (53-year-old, Latino) |  |  |
|  | There's that whole [issue,] you're not allowed to see your provider if you're not in the same state that they are licensed to practice in. And so there's a barrier there.   - Participant 18 (31-year-old, Black) |  |  |
|  | [They] can't provide services because you're out of state…I can't get services [here] in Oregon, because my insurance is in California.   - Participant 21 (31-year-old, Black Latino) |  |  |
|  | I do know its limitations. I think there's some structural limitations that need to be addressed at the policy level with insurance and being able to see patients across state lines because I travel a lot.   - Participant 17 (40-year-old, White) |  |  |
| Has view of telehealth changed since you began using? | I have a queer, non-binary mental health therapist, which I've never had the opportunity to have in my life, and it's made it so effortless to have a conversation…and it makes me feel so much more validated…It makes me feel so much safer.   - Participant 3 (32-year-old, White) | **View of telehealth: benefits** | - **Access to a pool of clinicians and the ability to access at convenience are selling points.** - **Useful for those with transport issues or busy schedules.** |
|  | That I know I can get a hold of a doctor is super helpful, just for my wellbeing…thinking I know someone's out there that I can reach. I'm much more willing to reach out to people and ask questions over the phone or on video.   - Participant 17 (40-year-old, White) |  |  |
|  | There are some gay guys that they are always doing stuff and if they feel sick at that moment [then] find[ing] a visit with a PCP is going to be a couple of weeks or a couple of months. So having someone there that…can prescribe some labs….[so you] can start treatment with antibiotics by telehealth [is helpful].   - Participant 1 (40-year-old, Latino) |  |  |
|  | …it's a resource. For a lot of people, including myself, doctors' offices are not ideal spaces. Nobody wants to sit in the doctor's office largely because they're sitting around people who are sick. I think it's a great middle ground to get the help you need without having to leave [home].   - Participant 5 (28-year-old, Black) |  |  |
|  | I started liking it more and more because I don't like having to call the doctor, set up an appointment, drive over there...it's much more convenient.   - Participant 12 (25-year-old, White) |  |  |
|  | Helps people connect with providers, maybe they didn't have the option to before, helps kind of expand what you have access to. And that's important for a lot of people who don't have the transportation, don't have those specialists in the close vicinity, or, maybe are stuck at home for whatever reason. It also provides opportunities for people who normally wouldn't want to see a counselor or don't have the ability to see a counselor or psychologist.   - Participant 2 (32-year-old, Asian) |  |  |
| How does telehealth impact health outcomes? | They put you on a waiting list for the next provider to see you. Which should be the way that it should be…if I feel bad at this moment, I need to see what is going on with me [now/immediately].   - Participant 1 (40-year-old, Latino) |  |  |
|  | If I can have someone that can at least tell me what I have to do, if I have to go to an urgent care or ER, or they can prescribe something that I can get from the pharmacy, that [is] amazing…I have a life that is really, really fast paced, and I don't have time to go to the urgent care and be there for a couple of hours to waiting to be seen when someone can [help me]…on the phone or on the camera.   - Participant 1 (40-year-old, Latino) |  |  |
|  | It could allow somebody [to]….get access to care like PrEP or other things that they maybe are too embarrassed to go to their family doctor to see about getting, and so it could provide an a more accessibility. To…feel more confident, and [use] their visit to get the things that they need for their own health that they're too, embarrassed to ask about.   - Participant 11 (44-year-old, White) |  |  |
|  | It's a great way to like establish a relationship with the doctor, because you feel more comfortable [in] your own home versus a clinical setting, and you're probably more likely to open up more with your doctor and talk about things that might be a little more sensitive.   - Participant 7 (40-year-old, Latino) |  |  |
| Which conditions are most or least easily addressed over telehealth? | If it's my endocrinologist that only needs to see a lab…Well, [they] can [order] from the computer and give me a phone or a video call and tell me this is what it is, so this is what we're going to do [for my health issue].   - Participant 1 (40-year-old, Latino) | **View of telehealth: preference for care** | - **Management of ongoing conditions, of quick flare ups of acute illnesses may be most suitable for telehealth.** |
|  | Refills, mental health, I would say...It could be headaches, or something like things that are internal, but that they can't necessarily figure out by touching you or listening to you.   - Participant 7 (40-year-old, Latino) |  |  |
|  | Something where I want to spend time talking to my provider, whether it's something related to mental health, or some type of skin condition where I'd rather be in person to show the doctor. Or if it's something that I feel is a little bit more serious than I'd prefer to go in person.   - Participant 2 (32-year-old, Asian) |  |  |
|  | I don't think it makes sense to use telehealth when you're doing physical therapy, or something that requires more movement…I just don't see how you could get that same level of level of motivation and direction on telehealth versus in person.   - Participant 2 (32-year-old, Asian) |  |  |
|  | For example, I sprained my ankle. Having a telehealth visit [wasn’t] beneficial for me, because the doctor needs to physically feel the feet. Whereas something like dermatology might look different. Because, taking a picture of the face, or whatever part of the body and having a doctor to look at it, they may be able to. I do think that it really is dependent on the scope of service.   - Interview 5 (28-year-old, Black) |  |  |
|  | If you're in bed, you know you can still seek the care that you need without having to get up and get dressed and go to the doctor's office, and you can still get your prescriptions filled, and then have someone go pick it up for you. It's a great use of current technologies.   - Participant 7 (40-year-old, Latino) |  |  |
|  | ...telehealth is for anything that's routine. If you're going in for hormones there's blood work that's sometimes done but the rest should be a little bit more of a routine process during quick checkups...it’s not really something that you need to have too much of a visual representation. You can update them on how you're feeling and stuff, so that [has] worked out perfectly over telehealth for me.   - Participant 20 (25-year-old, Latine) |  |  |
|  | I've had an STD. It’s easier for me to talk about like sex life stuff over the phone.   - Participant 17 (40-year-old, White) |  |  |
| Recommendations for improvement? | If we have an 11:30AM appointment. I connect to Zoom. And you know I just leave it on in the background. But he's connecting 20 min later. So that would be my only [recommendation].   - Participant 5 (28-year-old, Black) | **Improvement opportunities: Systems** | - **Ensure that systems have adequate plans for work flow and provider preparedness for engagement** - **Work with systems to offer physical telehealth service tools (i.e. smart devices, safe spaces for calls, pre-visit guides).** |
|  | I met this [new] doctor [on] telehealth for the first time, and I felt incredibly taken care of, I felt seen. It was thorough, and she was very warm. So just goes to show you, even telehealth can be better than in person, even from a human, personal [standpoint].   - Participant 7 (40-year-old, Latino) |  |  |
|  | I think it's more so about matching the provider, like the appropriate provider with what the needs are for the patient or client.   - Participant 14 (28-year-old, Black) |  |  |
| Recommendations for the future? | ….maybe skills of the therapist which is ensuring that your client feels heard or respected, and that the client feels trust in the provider, and that they have the skills appropriate to handle what you're talking about   - Participant 14 (28-year-old, Black) |  |  |
| Recommendations for improvement? | Those in need that do not have a phone, or device that can help them get connected to the internet, maybe provide that so it is easier for them.   - Participant 8 (38-year-old, Black) |  |  |
|  | That they know how to use the tool...know how to be present with the patient, that they know [the patient] has internet access, and that their background is not noisy.   - Participant 9 (33-year-old, Latino) |  |  |
|  | A better directory of LGBT friendly doctors that I can connect to easily. Just having some kind of way of filtering that out…these are people who are comfortable talking about gay issues.   - Participant 17 (40-year-old, White) |  |  |
| Recommendations for the future? | Not everyone has access to a laptop or a cell phone so possibly places where someone could go to have a telehealth appointment that's still private, but is available to everyone.   - Participant 11 (44-year-old, White) |  |  |
| Recommendations for improvement? | For employers, patients who are lower on the economic scale…allow them more access to [telehealth], if you know effective medical treatment [is available].   - Participant 3 (32-year-old, White) | **Improvement opportunities: Policy** | - **Policy level changes to cost through insurance or similar, and access can open doors for users.** |
|  | I think it's a universal problem in health care where certain doctors are considered in or out of network. And that makes people have to change doctors frequently when they might prefer to stay with the same one. If there were just one network, it would be a lot simpler.   - Participant 12 (25-year-old, White) |  |  |
| What would make telehealth more attractive? | If it was a more cost affordable option, people would definitely be way more intrigued   - Participant 20 (25-year-old, Latino) |  |  |
| What would make telehealth more attractive? | If there were organizations [advertised] that are specifically dedicated to LGBT health care, where you can go and schedule some tests or talk [with] someone about PrEP, that would probably make [telehealth] more appealing.   - Participant 2 (32-year-old, Asian) | **Improvement opportunities: Offerings** | - **Expanding pool of LGBT-specific providers, and mental health service offerings can increase user engagement.** |
|  | If you don't have any doctors that [are] necessarily specialized in LGBT health care, or maybe you're in an area where there's stigma around being LGBT, being able to find a healthcare provider that's within the same state but is more comfortable with LGBT people. I think that would be helpful and making the participant a little bit more comfortable [with telehealth].   - Participant 3 (32-year-old, White) |  |  |
| Recommendations for improvement? | It's really important that people who need mental health care recognize that they have access or the ability to talk to someone and have an open dialogue [over telehealth] and that there are people that can talk about queer relationships and identities and complexities there as well.   - Participant 3 (32-year-old, White) |  |  |
|  | I would say personalization, because we're people that have dynamic issues. And to that point, my mental health provider allowed me the ability to align with [a clinician] with my identity. And a lot of [other] providers can be doing that.   - Participant 3 (32-year-old, White) |  |  |
|  | Having more providers that identify as like LGBT+, I think that would help.   - Participant 21 (31-year-old, Black Latino) |  |  |
| What would make telehealth more attractive? | I think, especially communities who may not have the same access [to healthcare] as other people. Meeting them [through] social media, but that could be [through] a bus or subway advertisement too.   - Participant 3 (32-year-old, White) | **Improvement opportunities: Marketing** | - **Consider new locations and modes for marketing, including phones/apps, community locations, bars, and events.** |
|  | I would assume [advertising trough] apps. I would also assume bars and clubs. Spaces like that where people can see it and engage with it. I've been standing at urinals at bars, and I see things. And I'm like, Hmm! I'm gonna check that out just because it's in my face.   - Participant 5 (28-year-old, Black) |  |  |
|  | A lot of what's being done with marketing is on some sort of social app, whether it's Facebook, Instagram, scruff, grinder. Having the advertisements there is what gets my attention, whether or not I act on them. But something that shows that this is available to you.   - Participant 11 (44-year-old, White) |  |  |
|  | Advertising, perhaps in communities with pamphlets, maybe community outreach, door to door. I think word of mouth [also].   - Participant 15 (43-year-old, White) |  |  |
|  | Marketing plays a very big part, because I don't think we, as a community, acknowledge what resources are out there for us, in terms of using telehealth for PrEP [or] for PEP visits and things like that. I recently went to the doctor to get a supplement. I don't know if that's something I can do through telehealth…giving a broad picture of what [telehealth] looks like could certainly help.   - Participant 5 (28-year-old, Black) |  |  |
| Recommendations for the future? | On the apps. I would probably try to incorporate [advertisements] there. Maybe even television ads on channels that are geared towards us.   - Participant 16 (39-year-old, Multiracial) |  |  |
|  | Through television and radio. To be a little more inclusive to the community…featuring a gay couple in their advertising.   - Participant 19 (53-year-old, White) |  |  |
|  | Not only telling them about it, but giv[ing] them the resources. Tell them you don't necessarily have to go into a doctor's office, which is the whole point of telehealth. You don't have to go into a doctor's office to get your prep because the way technology and is working right now; you send your blood works on a piece of paper, and your pee, and you're good. It is just as good and is very convenient.   - Participant 10 (43-year-old, Black) |  |  |
| What would make telehealth more attractive? | I've seen a couple of ads recently. [A mental health organization] had done a celebrity ad on Facebook. I don't know who it was, but it was a personal story…they're doing it right.   - Participant 3 (32-year-old, White) | **Improvement opportunities: Advertising** | - **Tailor advertisements to the community (I.e. include diverse themes and actor types), and emphasize safety and privacy of use (underscores psychological safety of telehealth).** |
|  | Showing diversity of our communities. Because it's not all 25-year-old white boys…and [also] show the diversity of what's offered by telehealth. Letting people know that it's not just for your primary care or not, just for PrEP.   - Participant 11 (44-year-old, White) |  |  |
|  | They wouldn't have to worry about any private information coming out or getting out, or anybody else overhearing. So yes, time saving, privacy and discretion would definitely be the top selling points.   - Participant 16 (39-year-old, Multiracial) |  |  |
|  | Emphasizing no judgment and the ease of access…you can actually get an appointment immediately or quickly.   - Participant 17 (40-year-old, White) |  |  |
|  | Letting people know that these are private and confidential services will help.   - Participant 11 (44-year-old, White) |  |  |
| Recommendations for the future? | If you have STD symptoms you would like to [keep] private and you don’t want anybody [to] see you at the doctor's office, [telehealth] is safe. So, [telehealth is the] way to be a little bit private about the treatment or the diagnoses.   - Participant 1 (40-year-old, Latino) |  |  |
|  | I would say [advertising with] someone like a content creator. Maybe less [interested] if it were a section of a video that was sponsored. But if there was a dedicated post to [telehealth] where they were talking about it on their own accord.   - Participant 14 (28-year-old, Black) |  |  |
|  | I see a graphic of someone smiling while they're on their phone and you see the other side where the person is in their lab coat. You just see them enjoying the fact that they're able to utilize their mobile device in order to speak to their doctor.   - Participant 16 (39-year-old, Multiracial) |  |  |
| **Other Participant Quotations That Informed Themes** | | | |
| **Barriers To Use Theme** | There are times that we do have some problems. [Clinician] was trying to log in and then somehow couldn’t…I have found some technical problems with trying to use the telehealth.   - Participant 9 (33-year-old, Latino)   The only issue I had was that it was very time consuming. I wanted to make sure that whatever the organization that I did communicate with they were doing proper procedures and everything… it took me a little longer to figure out which one I wanted to go see.   - Participant 8 (38-year-old, Black)   If you don't fill out these forms and if you don't update your method of payment… they're threatening that they're going to cancel your appointment.   - Participant 6 (53-year-old, Latino)   I learned that you have to be very specific with your provider. You have to go in and tell them what you need.   - Participant 5 (28-year-old, Black)   My mental health provider had is based in California from my understanding and everything was set to pacific time. So that was initial challenge for me. And then my provider was just like, Oh, you can go with the app and update your time zone. And I was like, Oh, cool! I don't know if it was a default or something that had been updated and they didn't tell me.   - Participant 3 (32-year-old, White)   It's hard for me to get messages because I have to go into the platform and log in. And because of security they can't text to me, and text would be more accessible than having to go into the website   - Participant 3 (32-year-old, White)   There's been times where the technology is freezing up.   - Participant 21 (31-year-old, Black Latino)   The biggest thing is just [the video interface]. Sometimes there's connection problems. And I do think at one point in the [most recent visit] with her there might have been some connectivity issues.   - Participant 20 (25-year-old, Latine)   When the technology doesn't work properly, it does cause a little bit of a barrier or it kind of slows down the process.   - Participant 2 (32-year-old, Asian)   I was scheduled with a provider, and then they didn't show up.   - Participant 15 (43-year-old, White)   I mainly would say was an issue with trying to schedule, I guess it was an issue with timely scheduling per se.   - Participant 14 (28-year-old, Black)   And then, I think during the pandemic. I had a lot of issues where it would say, the provider would call from [app], and it would be like hours on end. But now that's kind of gone away. Because, you know, people aren't at home all the time.   - Participant 3 (32-year-old, White) | | |
| **View of telehealth: benefits theme** | They have messaging platforms. And so, if you don't think of something, or if you don't want to say it in that moment, you can message them on my chart.   - Participant 21 (31-year-old, Black Latino)   The provider that I was with at least definitely made sure that we would keep the communication going until we were, you know, satisfied with the appointment.   - Participant 20 (25-year-old, Latine)   Maybe you're disabled, it makes it hard for you to move or you just don't have the transportation to get there. I think having access to a doctor to help kind of resolve those needs, obviously, will allow you to get the medication you need or talk to a specialist about the problems that you're having.   - Participant 2 (32-year-old, Asian)   I think it should have a huge impact on [queer] men and anybody that don't like to go to the doctor.   - Participant 13 (45-year-old, White)   So I guess I would mention [telehealth works for] conditions that people are usually get treated for too late. But that genre of people probably having had heart attacks. Getting a colonoscopy, or maybe like chronic diseases. You know that we just catch too late, because we don't go to the doctor enough.   - Participant 13 (34-year-old, White)   I think, for people that are in are physically located in places that may otherwise not have access, it could be of great use to them.   - Participant 14 (28-year-old, Black)   I think maybe for behavioral mental health visits.   - Participant 15 (43-year-old, White)   I do see it more so as a very convenient tool.   - Participant 16 (39-year-old, Multiracial)   Telehealth is a lot easier to access for a lot of people who aren't able to like, just get out and go wherever. I really do appreciate the accessibility of telehealth.   - Participant 18 (31-year-old, Black)   This might be something that really is able to benefit somebody who is either disabled or you know, or for some other reason. Maybe you know, maybe they can't afford to get to the doctor.   - Participant 19 (53-year-old, White)   Helps people connect with providers, maybe they didn't have the option to before, helps expand what you have access to.   - Participant 2 (32-year-old, Asian)   Yeah, it's convenience. Like something where I can easily have someone that is license for provide a prescription. So that I can feel better as soon as possible.   - Participant 3 (32-year-old, White)   [Telehealth] also provides opportunities for people who normally wouldn't want to see a counselor or don't have the ability to see a counselor or psychologist.   - Participant 3 (32-year-old, White)   I feel like telehealth is sufficient, and saves a lot of time having to travel and wait in a waiting room, be exposed to other people who are sick.   - Participant 7 (40-year-old, Latino)   It will drastically improve everybody's access. It's just more access; more widespread access, easier access. It makes it extremely easier for people, you know, no matter where they are located.   - Participant 8 (38-year-old, Black) | | |
| **View of telehealth: preference for care theme** | Its [telehealth] there to attend minor conditions like those like for example, I call the flu or maybe pain that can be managed by from home or something that does not need lab or X rays.   - Participant 1 (40-year-old, Latino)   Just to treat small, small kinds of diseases in my case, and in that [recent] moment it was a sinus infection.   - Participant 1 (40-year-old, Latino)   Therapy mental health [as reason for use].   - Participant 10 (43-year-old, Black)   You know I primarily use it now for mental health. It's easy, because I don't have to be limited [in terms of access].   - Participant 3 (32-year-old, White)   I think basic nonthreatening things would be great for telehealth, like skin conditions. Maybe you have a cold, something that is contagious that you don't want to be around other people. I think things like that would be great for telehealth.   - Participant 11 (44-year-old, White)   It's very applicable for infections, if you are sick and you are experiencing flu or cold symptoms, you don't really want to go out, you're tired, you don't really want to drive, you don't really want to go interact with people and potentially get them sick.   - Participant 12 (25-year-old, White)   I think least applicable would be anything that would require a physical evaluation…I think most applicable would be probably maybe routine visits that maybe don't necessarily need to be done in office.   - Participant 14 (28-year-old, Black)   I think it works well for medication follow ups…it has been good for urgent care visits for me.   - Participant 15 (43-year-old, White)   Something that's easy to diagnose such as pink eye, or maybe the common cold.   - Participant 16 (39-year-old, Multiracial)   Anything that's gonna need like a physical examination, or like a, you know a laboratory sample or visual inspection that can't be done over a camera. It's best to go in person.   - Participant 17 (40-year-old, White)   Anything that's routine.   - Participant 20 (25-year-old, Latine)   Follow up care like medication management and follow-up care.   - Participant 21 (31-year-old, Black Latino)   I'm working from home, I can easily do a telehealth visits while I'm at home for 10 hours rather than do it on a day off, or take PTO, so again. It'll just, in my opinion, increase access to care.   - Participant 3 (32-year-old, White)   Still use telehealth in the sense that I get my prep prescription through it. So that's something that I'm using it for now.   - Participant 12 (25-year-old, White)   Like right now this afternoon, I have an appointment with my dietitian and she's going to be online.   - Participant 1 (40-year-old, Latino)   I see my primary care. We talk scheduled labs, we do everything, you know. It’s made it so easy because anything I needed she would just mail them out to me, like if I need swabs or anything. She'd mail them those out to me, and I'll just, you know, drop those off at a lab.   - Participant 10 (43-year-old, Black)   Just for primary care. Checkups, you know, every 6 months having that conversation, how everything's going, checking up on meds.   - Participant 11 (44-year-old, White)   I got stung by a stingray. I just called a video chat with my doctor and showed her the wound, and then she told me what to do, so that was good. I had the emergency, I was able to connect like immediately through the app, and I didn't have to go sit forever in a waiting room bleeding all over the place.   - Participant 17 (40-year-old, White)   Follow up visits if I have to have like a visit with the doctor to get like a refill. I prefer to meet online versus having to go to the doctor's office, for you know, something that's just essentially a conversation. Also I've used it for random concerns, like symptomatic sore throat or abdomen discomfort, you know, stuff where I just wanted to get like a quicker access to meeting with the doctor.   - Participant 7 (40-year-old, Latino) | | |
| **Improvement opportunities: Systems theme** | One of my teledoc visits. I just felt like the clinician or the nurse practitioner was being a little bit quick, you know, like rushing me.   - Participant 10 (43-year-old, Black)   Hopefully, in the future it [telehealth] advances even further, even more so that it's more accessible to more people.   - Participant 19 (53-year-old, White)   My time slot seemed short…especially with counseling. I never really feel like I'm able to complete everything I'm talking or want to talk about [due to time constraints]. So that's the only negative there.   - Participant 11 (44-year-old, White)   Maybe making sure that there's like good internet connection. And the doctor knows how to communicate, and stuff like that.   - Participant 13 (45-year-old, White)   It would be helpful if during telehealth visits, you could get those forms sent to you and just take them to whatever lab you need to or to whatever pharmacy you need to.   - Participant 2 (32-year-old, Asian)   Continue building up the technology. I mean making it easier, faster.   - Participant 8 (38-year-old, Black) | | |
| **Improvement opportunities: Policy theme** | Access to care is really important for folks…for telehealth, I think that would be…a cost reduction.   - Participant 3 (32-year-old, White)   The insurance company did not see it that way, and do not want it to be a situation where I pick them up every 3 months. They would rather bill for it every month. It's [access issues] mostly with the insurance company.   - Participant 19 (53-year-old, White)   Unfortunately, I don't have access. It's not covered and due to my illness…Based on [blank] regulations.   - Participant 3 (32-year-old, White)   I went to like a tele-therapist for 5 sessions and it was fine like it was. The visits themselves were fine. It was just a lot of red tape and bureaucracy around it, it ended up being really obnoxious and problematic.   - Participant 17 (40-year-old, White)   I think one of the biggest things is making sure that it's at a cost level, that even if you don't have insurance that you can still be covered easily. That would to me would be a great way to keep it accessible for everyone.   - Participant 11 (44-year-old, White)   I know a lot of people who don't have health insurance. So that could be putting them off of it. Because if they don't have health insurance that covers telehealth, then it's not an attractive option for them, because then they have to pay for it [out of pocket].   - Participant 12 (25-year-old, White)   I think just making it easier is always going to be the most attractive. Just the ease of use and dealing with insurance and providers, and all of that.   - Participant 17 (40-year-old, White)   Not everyone has health insurance or can afford to go to the doctor. So maybe if there was lower [cost], free options through some type of program, that would make it more appealing to a lot of people.   - Participant 2 (32-year-old, Asian)   Making it affordable for those who don't have insurance [is a recommendation].   - Participant 7 (40-year-old, Latino)   If telehealth is by default 50% cheaper…might be a way to bring more people.   - Participant 9 (33-year-old, Latino) | | |
| **Improvement opportunities: Offerings theme** | I would say providers that have maybe like a variety of skill sets, so for therapy I know there's like emdr certification or therapists are more tuned with specific problems. And I felt like a lot of times the therapist like, I found online we're just general therapy and it wasn't specific.   - Participant 14 (28-year-old, Black)   Having direct access to providers that specialize in it [LGBT care]. Like, for example, in the ___ area, there's this whole center called the _____ clinic, and you know, they specialize a lot in LGBT care.   - Participant 2 (32-year-old, Asian)   Having more equipment available to the patient [would make telehealth more attractive].   - Participant 21 (31-year-old, Black Latino)   Mental and health related counseling. If they [all] were to offer that, I think that that is huge. That would attract a lot of people.   - Participant 8 (38-year-old, Black) | | |
| **Improvement opportunities: Marketing theme** | We have all of these resources. Social media. Now being one of the biggest, because that's where I get my news from.   - Participant 10 (43-year-old, Black)   I don't think I've ever seen a like telehealth billboard, I don't think I've seen any, like, advertising for one that wasn't associated with the health insurance company. I feel like it would be good if it was [marketed this way].   - Participant 12 (25-year-old, White)   Another thing that would make it attractive is…just seeing other people that you know use it. Maybe introducing it to people when they are at already at a doctor's office or in the hospital.   - Participant 13 (45-year-old, White)   Word of mouth or [new] advertising.   - Participant 15 (43-year-old, White)   Gay bars have those pamphlets all the time. I don't know just taking out an ad in those would be good.   - Participant 18 (31-year-old, Black)   We're stuck in social media culture. And it [marketing] should be meeting users, meeting clients, and patients where they are and making sure that it's like tailored towards their needs.   - Participant 3 (32-year-old, White) | | |
| **Improvement opportunities: Advertising Theme** | Now you have all these online stores popping up all over social media and stuff. So it's very easy now for people to just flip through their stories or flip through their reels and see an ad and say, Hey, you know, if you want this, you know, this is how.   - Participant 10 (43-year-old, Black)   I think the privacy [is a selling point].   - Participant 1 (40-year-old, Latino)   It all goes back to education. The thing is that a lot of people don't even know that when it comes to telehealth and PrEP, that they have resources, that they can get the meds for free, or subsidized. So yeah, that’s definitely how to glamorize it. You have to push it to the people or the gays who don't already know about it.   - Participant 10 (43-year-old, Black)   The convenience and that you don't have to wait for an appointment. The convenience of doing it at home and the speed of being able to just instantly be seen if there's someone available.   - Participant 12 (25-year-old, White)   Just getting real faces out there to show people that telehealth can work for a lot of people.   - Participant 15 (43-year-old, White)   And also, it's not just video. You can just do audio. You can text, you know, like there's so many means of communication where you could just feel like more private, or you're safer and more comfortable.   - Participant 7 (40-year-old, Latino)   I would promote the fact that you can meet a doctor anywhere, anytime, so like you don't have to leave work. You don't have to leave home. You don't have to worry about spreading germs or catching germs.   - Participant 7 (40-year-old, Latino) | | |
| **Psychological Safety Theme** | It can be annoying to if you don't have the option to see the same one [clinician], again, to have to reintroduce yourself and re-explain everything to another person, you know?   - Participant 12 (25-year-old, White)   I've just been more comfortable with the different [clinician and access] options that are available with telehealth   - Participant 15 (43-year-old, White)   Ever since COVID I’ve had really bad anxiety around being in places that that I'm not familiar with. I like that I can actually talk to my doctor about things that I need to talk to her about.   - Participant 4 (26-year-old, Biracial)   I think for me, the most important thing is the relationship I have with the actual medical doctor.   - Participant 6 (53-year-old, Latino)   [Telehealth] is a good experience, just communicating with the doctor, because everything that I needed and regards to the hormones is communicated.   - Participant 20 (25-year-old, Latine)   And just also being able to talk to providers that are also part of the LGBT community. And I know a lot of people also feel comfortable that as POC, they are speaking with POC doctors.   - Participant 2 (32-year-old, Asian) | | |
